# Simple Assumptions to Improve Markov Illuminance and Reflectance

**DOI:** 10.3389/fpsyg.2022.915672

**Published:** 2022-07-08

**Authors:** Yuki Kobayashi, Akiyoshi Kitaoka

**Affiliations:** ^1^Research Organization of Open Innovation and Collaboration, Ritsumeikan University, Ibaraki, Japan; ^2^Japan Society for the Promotion of Science, Tokyo, Japan; ^3^College of Comprehensive Psychology, Ritsumeikan University, Ibaraki, Japan

**Keywords:** lightness/brightness, computational model, illusion, Markov random field, Bayesian model

## Abstract

Murray recently introduced a novel computational lightness model, Markov illuminance and reflectance (MIR). MIR is a promising new approach that simulates human lightness processing using a conditional random field (CRF) where natural-scene statistics of reflectance and illumination are implemented. Although MIR can account for various lightness illusions and phenomena, it has limitations, such as the inability to predict reverse-contrast phenomena. In this study, we improved MIR performance by modifying its inference process, the prior on X-junctions, and that on general illumination changes. Our modified model improved predictions for Checkerboard assimilation, the simplified Checkershadow and its control figure, the influence of luminance noise, and White’s effect and its several variants. In particular, White’s effect is a partial reverse contrast that is challenging for computational models, so this improvement is a significant advance for the MIR framework. This study showed the high extensibility and potential of MIR, which shows the promise for further sophistication.

## Introduction

In lightness/brightness perception research, it is desirable to establish a computational model that precisely predicts human perception (e.g., Kingdom, 2011; Shapiro and Lu, 2011). Although Gestalt theories have made many contributions to the development of this research field (e.g., Gilchrist et al., 1999; Bressan, 2006), computational models (here, we refer to models that output rigorous predictions of human perceptions of any arbitrary image inputs) have also been discussed by many researchers. For instance, spatial-filtering models are known as a promising approach that many researchers have examined and discussed (Blakeslee and McCourt, 1997, 1999; Dakin and Bex, 2003; Economou et al., 2007; Robinson et al., 2007; Betz et al., 2015; Zeman et al., 2015).

Although spatial-filtering models are known to have high performance for brightness prediction (e.g., Blakeslee and McCourt, 2012), it is not necessarily easy to interpret psychological implications from each computational process in these models (e.g., to assign high weights to higher frequency filter outputs). This is not surprising because they are motivated by physiological processes in the human brain (Blakeslee and McCourt, 1999; Robinson et al., 2007) and aim to account for low-level processing in brightness perception (Blakeslee and McCourt, 2012). However, if there is a computational model whose processes and structures are intuitively understandable as a representation of the human mind, it will be useful for quantitative output predictions for arbitrary images and contribute to the qualitative explanation of the perception of lightness/brightness.

Murray (2020)’s novel lightness model, Markov illuminance and reflectance (MIR), is promising as an understandable computational model. MIR is based on a probabilistic model called a conditional random field (CRF). MIR’s CRF design is composed of some prior assumptions motivated by natural-scene statistics (e.g., “Illuminance spans a wide range, and lower illuminances are more likely”; Murray, 2020, p. 19), supposing that humans utilize these statistics to solve lightness ambiguity (Allred and Brainard, 2013; Murray, 2013; Feldman, 2015). Therefore, large parts of the computational processes in MIR have a certain amount of psychological rationality, and the meaning of each process can be interpreted relatively straightforwardly. In MIR, the model output is obtained as an illuminance map (perceived illumination, represented by a lux unit) and a reflectance map (perceived reflectance, or lightness, represented by a percent unit), and thus each output value is less arbitrary, unlike the outputs of spatial-filtering models. Murray (2020) also argued that MIR is compatible with the widely known Gestalt theories (Gilchrist et al., 1999) because both share the idea that general lightness perception can be explained by a relatively small number of principles. Indeed, MIR incorporates Gestalt ideas into the CRF, such as “illuminance edges tend to be straighter than reflectance edges” (MIR’s constraint A7) (Adelson, 2000) or “illuminance changes tend to be gradual, not abrupt” (MIR’s constraint A3) (Land and McCann, 1971; Agostini and Galmonte, 2002b). MIR has been reported to be able to account for many lightness illusions and phenomena, suggesting a high probability that it will be a novel pathway in this research field.

In this study, we aimed to extend MIR. We present a modified version of MIR that can account for different types of lightness phenomena. The current version of MIR (Murray, 2020) does not predict reverse contrasts (Nedimović et al., 2021), where target areas neighboring dark regions appear darker than an equiluminant target neighboring bright regions (Bressan, 2001; Agostini and Galmonte, 2002a; Economou et al., 2015). White’s effect is one of the most famous reverse contrasts (White, 1981, 1979). This is a partial reverse contrast in which a gray bar that shares its longer edge with a dark adjacent area appears darker than an equiluminant gray bar that shares its longer edge with a bright adjacent area. Reverse-contrast phenomena are challenging and important issues for lightness/brightness models to explain because they highlight the fact that lightness/brightness perception is more complicated than simply being determined by immediate contrast (Gilchrist, 2006; Economou et al., 2015; Agostini et al., 2020). It is a large leap for computational models to be able to explain White’s effect, as suggested by the fact that the ODOG model, a famous spatial-filtering model (Blakeslee and McCourt, 1999), showed its high performance by accounting for White’s effect (see Betz et al., 2015 for a counterargument), which the authors’ preceding model (the DOG model) could not account for (Blakeslee and McCourt, 1997).

We modified some prior assumptions of the original MIR to reflect natural scenes better and improve its performance. The most important improvement was the successful prediction of White’s effect, but our model also includes several improvements. Note that we designed the proposed model through multiple performance tests. We have made our Python and Julia codes available for readers at https://osf.io/ank4r/, and we welcome further tests, improvements, and discussions (see the Section 1 in the Supplementary Material).

## Modifications to the Original Model

### Full Use of the Available Links Between Nodes

First, we modified the original MIR’s belief-propagation schedule. This is a technical modification of the model’s inference process rather than a modification of the model’s priors. Although the original belief propagation efficiently converges to approximate solutions, it does not fully utilize the available links in the CRF. We added another phase of message passing to utilize all the links in the CRF, and this helped the model to search for a solution more efficiently. See the Section 2 in the Supplementary Material for details of this modification.

### Prior 1: Sign-Invariant Edges in X-Junctions Cue Illumination Changes, but Sign-Variant Edges Do Not

The original MIR considers that “X-junctions are evidence for illuminance edges” (Murray, 2020, p. 20) and assigns no cost to the illuminance change between two pixels that constitute an X-junction. This rule contributes to the correct predictions of illusions that give shadow impressions, such as the Argyle illusion, Snake illusion, and Koffka ring (Adelson, 2000). In the original MIR, this rule was applied to any X-junction without considering the relationships among the luminances of the four pixels.

**FIGURE 1:**
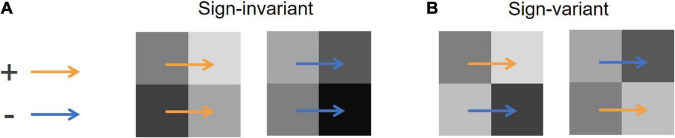
Examples of X-junctions. Each set of four squares represents an X-junction composed of four pixels. The orange arrows indicate an increasing edge, where a pixel at the arrowhead is brighter than that at the arrow’s root. The blue arrows indicate a decreasing edge, where a pixel at the arrowhead is darker. **(A)** Examples of sign-invariant edges in an X-junction. Two vertical colinear luminance edges (those between two horizontally aligned pixels) are both increasing (left) or decreasing (right). **(B)** Examples of sign-variant edges in an X-junction. Two vertical colinear luminance edges show inconsistent contrast polarities.

Although X-junctions are likely to occur in situations where illumination boundaries exist, they do not always do so. When two colinear luminance edges in an X-junction show the same contrast polarity (Figure 1A), these edges can be considered a cue of illumination changes. However, when two colinear luminance edges in an X-junction show different contrast polarities (Figure 1B), they are unlikely to cue an illumination change. Following Gilchrist et al. (1983) and Gilchrist (2014), we call these two types of edges “sign-invariant” and “sign-variant” edges, respectively. If two colinear luminance edges are only caused by an illumination change, the edges must be sign-invariant. A white–black checkerboard that is uniformly illuminated, for example, consists of many X-junctions caused only by reflectance edges (all of them are sign-variant edges), but the original MIR is likely to see them as illumination changes. The model’s expectation for illumination changes at a pixel pair is represented by a cost parameter assigned to the pair; a high cost (*w*) indicates the model’s lower expectation for illumination changes (i.e., uniform illumination). We put a higher cost on sign-variant edges in X-junctions (*w*_*Xvar*_ = 50) than on luminance edges not included in X-junctions, to which a cost ranging between 0 and 50 is assigned, as described in the next section. This parameter design indicates the model’s prior that illumination changes are less likely at sign-variant edges in X-junctions than at luminance edges not included in X-junctions. We also kept the cost on sign-invariant edges in X-junctions at zero (*w*_*Xinv*_ = 0) and the cost on equiluminant pixel pairs at 600 (*w*_0_ = 600), as in Murray (2020). It is widely known that humans take into account contrast polarities in X-junctions when they judge surface characteristics (Kitaoka, 2005), and X-junctions are presumably important for solving the lightness–illumination ambiguity (e.g., Adelson, 2000), so the complication of this prior seems necessary for model improvement.

### Prior 2: Scenes With More Luminance Contrasts Contain More Illuminance Changes

In the original MIR, the cost for illuminance changes in two pixels at a luminance edge (except for two pixels included in an X-junction) was held constant [the weight for Equations 6 and 7 in Murray (2020) was held at *w*_1_ = 20]. This parameter largely controls the contrast of the model’s illuminance map output. Holding it constant means that the model does not modulate its expectation regarding illumination changes for any images or scenes.

However, information included in an entire image is useful for judging whether a certain luminance edge should be attributed to illumination or reflectance. A scene with high illuminance contrast (i.e., illumination that is less homogeneous) also casts an image with a high luminance contrast. Therefore, it is plausible to expect more illuminance change for an image with a high luminance contrast. This assumption may seem too simple, but we observed that it plays a good role in predicting many illusions (see section “Model Performance”). We implemented this assumption into the model by modulating the parameter *w_1_* depending on the luminance contrast of the input image.

In our modified model, parameter *w_1_* was controlled as below. First, as a measurement of an image’s contrast, the average Michelson contrast can be obtained as:


$$M\,C_{t\,o\,t\,a\,l}=\sum_{i}\left(\frac{\left|l_{i\,1}-l_{i\,2}\right|}{l_{i\,1}+l_{i\,2}}\right)$$

$$M\,C_{a\,v\,e\,r\,a\,g\,e}=\frac{M\,C_{t\,o\,t\,a\,l}}{\sqrt{m^{2}+n^{2}}}$$

where *i* represents each pair of adjacent pixels in the input image, and *l*_*i1*_ and *l*_*i2*_ are luminance levels (cd/m^2^) of the two pixels constituting *i*. Therefore, *MC*_*total*_ is the sum of the Michelson contrast of all adjacent pixel pairs. The image’s height and width (numbers of pixels) are represented by *m* and *n*, respectively, so the denominator of the second equation is the length of a diagonal line in the image. *MC*_*average*_ is used as a measurement of an image’s luminance contrast (here, the term “average” is not used in the strict sense). *MC*_*total*_ or that quantity divided by the number of pixels may seem to be useful measurements but they vary depending on the image size (i.e., these values increase or decrease when an image is up-sampled). *MC*_*agerage*_ depends on both the luminance range and the magnitude of articulation in a stimulus image. The parameter *w_1_* is determined as *w*_1_=*w*_*Xvar*_×exp(−*MC*_*average*_). This exponential function returns 50 (*w*_*Xvar*_) when an image has a zero luminance contrast (i.e., a homogeneous image), and the returned value decreases as the image’s luminance contrast increases, but it never reaches zero. Therefore, *w_1_* is automatically determined by the image content, unlike Murray (2020)’s original model, and it falls in the range between *w*_*Xinv*_ and *w*_*Xvar*_.

## Model Performance

Below, we demonstrate the performance of our modified model by comparing it with Murray (2020)’s original model. One run consisted of five iterations in Murray (2020)’s original implementation, but one iteration in our modified model requires twice as much computation as the original because of the modification of the belief propagation schedule. Therefore, in the following model tests, one run of the modified model consisted of three iterations and that of the original model consisted of six. This makes the model comparison fairer because it makes single runs of both models equally computationally complex.

### Main Illusory Images Employed by Murray

**FIGURE 2:**
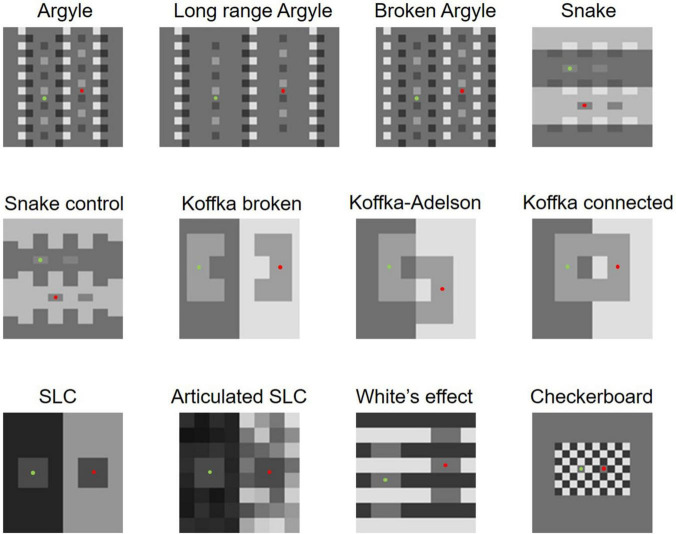
Twelve stimuli used by Murray (2020). Green and red dots indicate the target areas.

Murray (2020) employed 12 famous illusory figures to examine the model’s performance (Figure 2). First, we compared the two models’ performances using them. Murray (2020) adopted the best result (with the lowest energy) of 10 runs, but we observed minor random fluctuations in the outputs with this number of runs; thus, we decided to take the best result of 30 runs to improve the accuracy.

Table 1 shows the predictions of the two models for all 12 images. They are similar, except for White’s effect and Checkerboard assimilation, which we will discuss later. The modified model predicts illusions for images where the original model also predicts illusions, and the modified model does not predict illusions for images where the original model does not. The modified model does not predict an illusion in Koffka connected while the original does, but Murray (2020)’s experiment on human observers indicated that this figure does not cause a significant illusory effect; thus, the modified model’s prediction may be closer to human perception. The modified model also successfully accounts for differences in illusion magnitudes in the stimuli pairs (Argyle vs. Broken Argyle, Snake vs. Snake control, Koffka–Adelson vs. Koffka connected, Koffka broken vs. Koffka connected, and SLC vs. Articulated SLC), for which the original model was reportedly able to account (Murray, 2020). Moreover, the modified model also predicts the effects of the highest luminance rule, glow, codetermination, and articulation as the original did (these results are shown at https://osf.io/ank4r/). Therefore, the modified model replicated all the successful results reported by the original model in Murray (2020).

**TABLE 1:** The modified (M) and original (O) models’ lightness (perceived reflectance) predictions for the target areas in the 12 figures employed by Murray (2020).

	Left (M)	Right (M)	Left (O)	Right (O)
Argyle	0.68	0.41	0.68	0.41
Long range Argyle	0.68	0.41	0.68	0.41
Broken Argyle	0.41	0.41	0.41	0.41
Snake	0.80	0.48	0.62	0.48
Snake control	0.54	0.54	0.54	0.54
Koffka Broken	0.66	0.51	0.66	0.51
Koffka–Adelson	0.66	0.51	0.74	0.51
Koffka connected	0.57	0.57	0.66	0.57
SLC	0.59	0.40	0.59	0.40
Articulated SLC	0.76	0.40	0.76	0.40
White’s effect	0.46	0.41	0.41	0.41
Checkerboard assimilation	0.41	0.41	0.36	0.68

*“Left” and “Right” refer to the target areas indicated in Figure 2 by green and red dots, respectively.*

**FIGURE 3:**
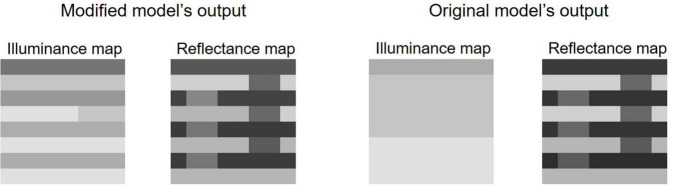
Outputs for the predictions of White’s effect by the two models.

For White’s effect and the Checkerboard assimilation, where the original model fails, we observed improvements in the modified model. The modified model correctly predicts White’s effect and sees clear illuminance stripes (Figure 3). We focused on the prediction of White’s effect in this study, and it will be discussed further in a later section.

The modified model also improved in the prediction of Checkerboard assimilation (De Valois and De Valois, 1990; Blakeslee and McCourt, 2004). It perceives the same lightness level for the two targets, whereas the original model sees the right target as much lighter. Humans perceive the left one slightly lighter (Murray, 2020), but the effect depends upon the frequency of the checks (Blakeslee and McCourt, 2004). The modified model’s prediction is obviously closer to human perception, although it may not be complete. This difference between the predictions by the two models is largely caused by the modification of the belief propagation schedule. Similarly, it also largely reduced the energies (i.e., the model finds a better solution) in the prediction for Articulated SLC. In summary, for the stimuli used by Murray (2020), the modified model provided several improvements without any performance deteriorations.

### Influence of Pixel-Wise Luminance Noise

**FIGURE 4:**
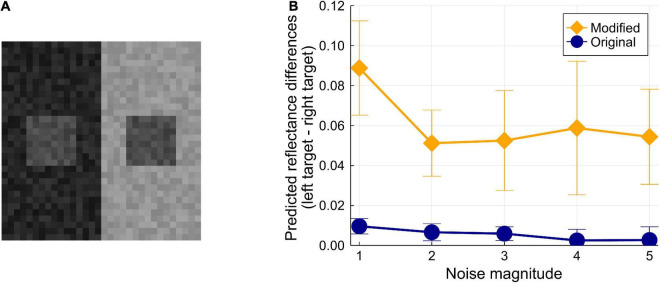
**(A)** Example of a noised image. In this example, the lower and upper limits of the uniform distribution are ± 5. The SLC effect should be present in most observation environments. **(B)** Results of the test for the noise’s influence on the model predictions. Each error bar represents the standard deviation of 10 tests in each condition.

Betz et al. (2015) examined the ODOG model (Blakeslee and McCourt, 1999) by running it on White-effect figures with narrowband noise. They found that the influences of narrowband noise on the ODOG model and on humans do not match. Testing models with noised images is important for investigating whether the correct prediction can be observed robustly. However, using narrowband noise is difficult in our case because the models assume smaller images; thus, we created images with pixel-wise luminance noise based on Murray (2020)’s SLC figure (Figure 2). First, the 16 × 16 image was up-sampled to be a 32 × 32 image so that the influence of each pixel’s noise was moderate. Then, all pixel luminances were independently noised; random and independent samples from a uniform distribution were added to each pixel (the distribution’s expected value was zero, and the upper and lower limits were manipulated). One of the two targets was copied onto the other target to make both targets physically identical. We prepared five conditions by manipulating the magnitude of noise (i.e., the upper and lower limits of the uniform distributions of the noise) from one to five. Although we did not conduct an experiment on human observers, our informal observation confirmed a stable illusory effect even for the SLC image with the most noise (Figure 4A).

We created 10 randomly noised images for each noise magnitude condition (one to five); thus, 50 images were obtained. The two models were tested on these images (here, a single test was composed of 10 runs, not 30 runs) (Figure 4B). The results revealed that the original model is vulnerable to pixel-wise noise; it predicts a very weak illusion even for an image with the smallest noise. However, the modified model correctly predicts moderate illusions consistently for all conditions. The modified model’s output also shows a trend: the illusion magnitudes decrease with larger noise, which human observers should observe as well. These results show that the original model’s correct prediction for SLC is less robust and that the modification to the model enhanced its stability against luminance noise. This improvement is mainly because of the modification of the belief propagation schedule.

### Simplified Checkershadow Illusion

**FIGURE 5:**
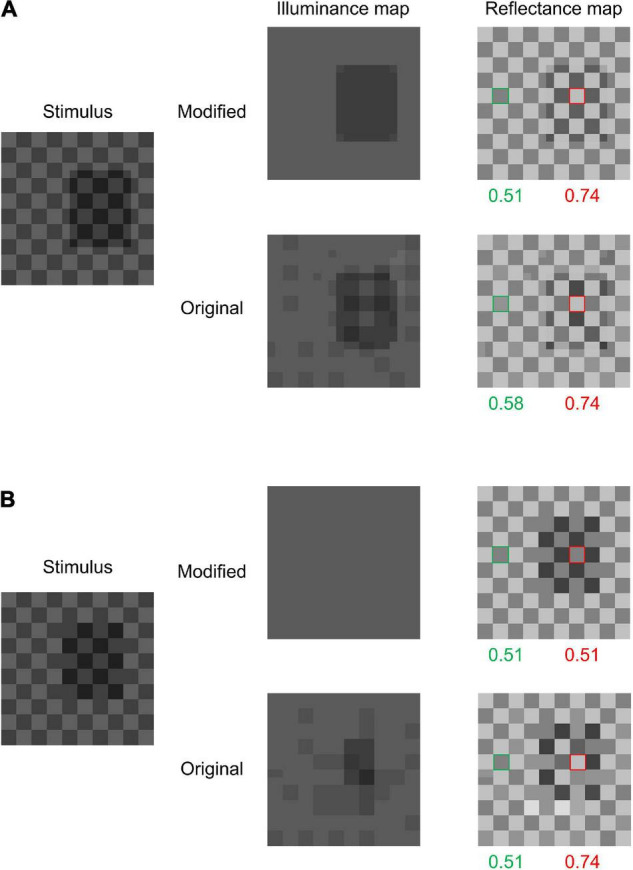
**(A)** Simplified Ceckershadow Illusion (SCS) and both model outputs. Both models perceive the difference between the two target area reflectances (tiles with green and red frames), but the modified model perceives the shadowed region more clearly. **(B)** The SCS control and the models’ outputs for it. The modified model does not observe a reflectance difference, while the original model does.

We tested the models on images composed of sign-variant and sign-invariant edges. We employed Blakeslee and McCourt’s (2012) (Fig. 3) because it can be represented using a small simple image. The image used in this study is shown in Figure 5A left. This image can be understood as a simplification of Adelson (1995)’s famous Checkershadow Illusion. As in Adelson (1995)’s original image, the figure is composed of a checkerboard and a simulated shadow (or transparent filter), and lighter tiles in the shadow have a luminance that is physically equal to that of darker tiles out of the shadow. However, humans perceive the former as lighter (and brighter) compared with the latter (Blakeslee and McCourt, 2012). However, the illusory effect becomes much weaker or absent when the shadow is shifted to share its boundary with edges in the checkerboard (Figure 5B left). We tested the two models on these two images (hereafter, we call them SCS, referring to the simplified Checkershadow Illusion, and SCS control, respectively).

Figure 5 shows the results of the tests. For the SCS, both models correctly predict illusory effects with similar magnitudes. However, the illuminance map of the original model contains speckled noise, which does not match our intuitive illumination impression. The modified model outputs an illuminance map that better matches the illumination impression by humans, containing a clear shadowed region. More importantly, the original model predicts an illusion for the SCS control image with a magnitude similar to (or stronger than) that for the SCS, while the modified model does not. Thus, the modified model correctly predicts the presence of the illusion for the SCS and its absence for the SCS control, while the original model predicts illusions for both. This difference in performance between the two models is obviously caused by the modification to the prior regarding X-junctions.

### Further Tests for White’s Effect

As mentioned, the modified model’s most important improvement is its ability to account for White’s effect, which has been an important and challenging issue for lightness models and theories (Anderson et al., 2001; Howe, 2005; Bressan, 2006). We further tested our model on other variants of the White-effect figure.

**FIGURE 6:**
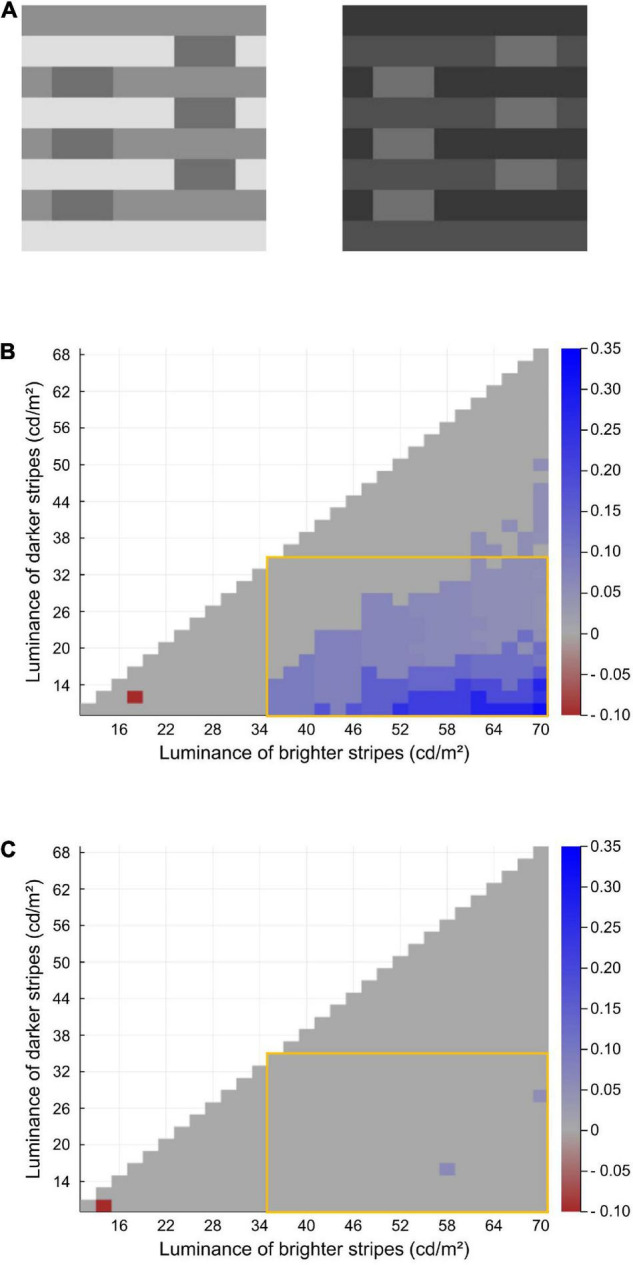
**(A)** Double-increment version (left) and double-decrement (right) version of the White-effect figure. Both are considered to cause virtually no illusions. **(B)** Heatmap of the modified model’s illusion prediction for the White-effect figure with various stripe luminance conditions. In the original image used in Murray (2020) and the test shown in Figure 3, the two stripes’ luminances were 17.5 and 70 cd/m^2^. **(C)** Heatmap of the original model’s prediction.

Spehar et al. (1995) reported that White’s effect becomes almost absent when both the two luminance levels of the stripes are modified either to be brighter (Figure 6A left) or darker (Figure 6A right) than the gray targets (i.e., the targets are in double decrements or double increments). We examined the model prediction by manipulating the luminance levels of the two surrounding stripes. In this test, both stripes’ luminances were manipulated from 10 to 70 cd/m^2^ with 2-cd/m^2^ intervals (i.e., the brighter ones’ luminance range from 12 to 70 cd/m^2^, and the darker ones’ range from 10 to 68 cd/m^2^). Conditions where the two stripes’ luminances become equal or reversed were not included. The target luminance was fixed to that used in Murray (2020)’s original White’s-effect stimulus, which was 35 cd/m^2^.

The results are shown in Figures 6B,C via heatmaps. Each row indicates the luminance levels of the darker stripes, and each column indicates those of the brighter stripes. The color of each block indicates the magnitude of the predicted illusion; i.e., the difference between the predicted lightness of the two targets (left–right). The modified model generally predicts an illusory effect when the target luminance lies between the two stripes (the area surrounded by the orange frame; Figure 6B) and predicts greater effects as the contrast between the stripes becomes larger. Moreover, the model rarely predicts an effect when the targets are in double increments or double decrements (outside of the orange frame; Figure 6B). These results show the robustness of the correct predictions for White’s effect and its control version (Spehar et al., 1995; Howe, 2005). Figure 6C shows the results of the same test on the original model. The original model’s performance for these stimuli is obviously worse than that of the modified one.

**FIGURE 7:**
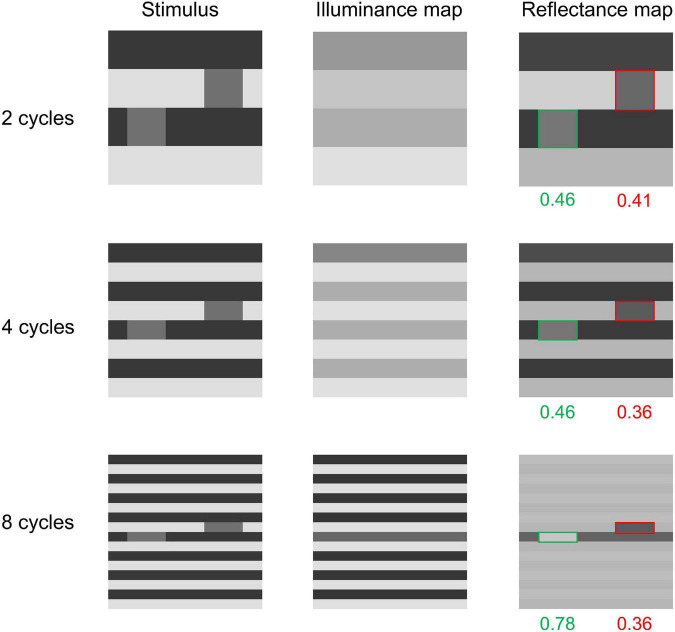
Effect of stripe frequency on the modified model’s output. The modified model predicts stronger illusions as the stripe frequency increases.

White’s effect is known to be enhanced when the stripe’s frequency becomes higher (White, 1979; Blakeslee and McCourt, 2004). This can be easily tested even on 16 × 16 sized images. We tested our model on three conditions (Figure 7) where the stripe’s frequencies were 2, 4, or 8 cycles/image. The luminance levels of each region were equal to those in Murray (2020)’s original figure (Figure 2). The results showed that the modified model correctly predicts a stronger effect for a figure with a higher stripe frequency (Figure 7). The original model does not predict illusions for any of these three conditions (the results of the original model are shown in the Section 3 in the Supplementary Material and the online repository).

**FIGURE 8:**
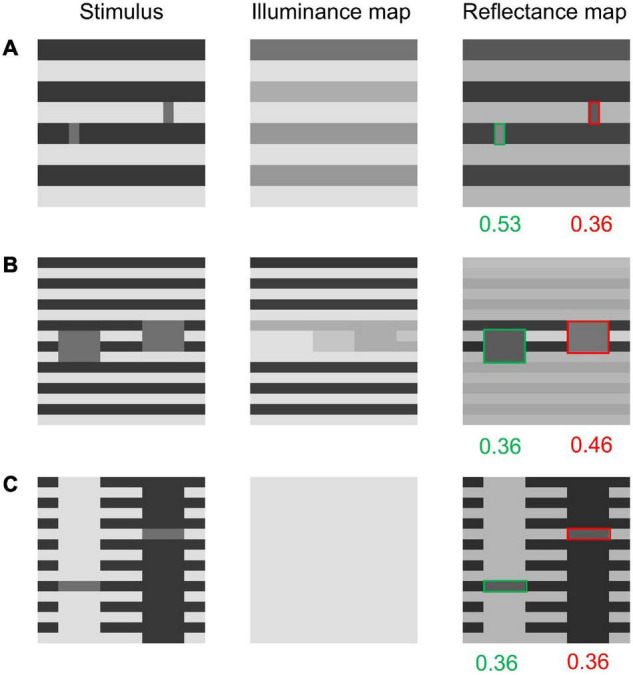
Some variants of the White-effect figure and the modified model’s outputs for them. **(A)** A White-effect figure variant with narrow targets. The model correctly predicts illusion enhancement (i.e., a stronger effect than in the original White-effect figure). **(B)** A White-effect figure with taller targets. The model predicts a reversal of the effect. **(C)** Howe (2001)’s variant. The modified model correctly predicts no illusion.

The modified model also succeeds in predicting human perceptions for some other variants of White’s effect. It has been suggested that White’s effect is enhanced as the target width (more specifically, the lengths of the sides parallel to the stripes) becomes shorter (Kingdom and Moulden, 1991; Blakeslee and McCourt, 2004). The modified model predicts a slight enhancement for narrower targets (Figure 8A). Moreover, Otazu et al. (2008) introduced a White-effect variant in which the effect is greatly reduced when targets span three stripes. Via informal observation, we observed that the effect was reversed rather than canceled, as shown in Figure 8B. Our model thus predicts an illusion reversal from White’s original effect. Furthermore, the model correctly predicts no illusion for Figure 8C, which was introduced by Howe (2001) as a control for White’s effect. Predicting the absence of the effect in this control figure is challenging for models that consider T-junctions around the targets as the main cause of White’s effect. The present model does not explicitly take into account T-junctions in its processing, and thus it correctly predicts the elimination of the effect. For the three White-effect variants discussed here, the original model does not predict any illusions (see the Section 3 in the Supplementary Material and the online repository for details).

### Limitations of the Modified Model

**FIGURE 9:**
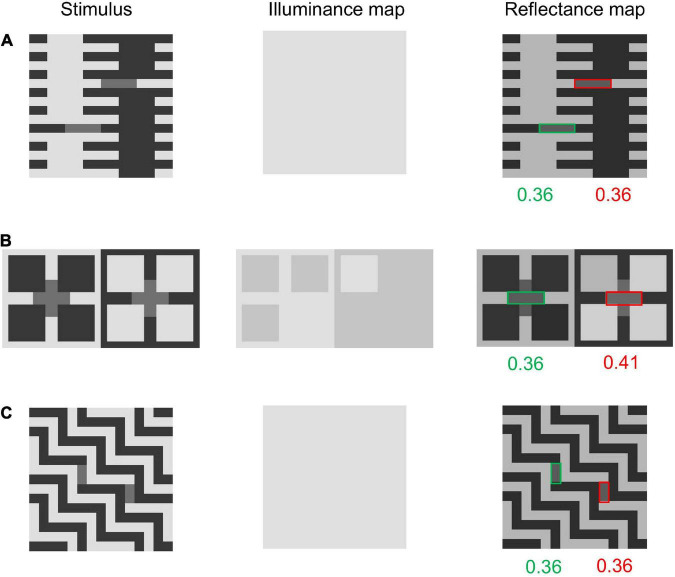
Variants of the White-effect figure and the modified model’s outputs for them. **(A)** Anderson et al. (2001)’s variant. The model does not predict an illusion for it. **(B)** Todorović (1997)’s variant. Although the model predicts an illusion for the case shown in this figure, it is highly unstable. **(C)** Clifford and Spehar (2003)’s variant. The modified model does not predict an illusion.

We have shown several successful predictions by our modified model, but we are also aware that it has some limitations. First, our model does not predict the illusory effect in Anderson et al. (2001)’s configuration (Figure 9A). Anderson et al. (2001) created this figure to show that White’s effect is recovered when targets are shifted from their positions in Howe (2001)’s control figure (Figure 8C). Thus, it is challenging for computational models to predict both the effect’s presence in Anderson et al. (2001)’s stimulus and the effect’s absence in Howe (2001)’s version. Spatial-filtering models also fail at this (Robinson et al., 2007). Moreover, our model does not predict a stable illusion for Todorović (1997)’s White-effect variant (Figure 9B). The model sometimes predicts the effect, as in the case shown in Figure 9B, but it is not stable (see the Section 3 in the Supplementary Material). Our model also does not predict an illusion in Clifford and Spehar (2003)’s zigzag variant of White’s effect (Figure 9C),^1^ which is reportedly accounted for by some spatial-filtering models with certain parameter settings (Robinson et al., 2007; Zeman et al., 2015). The original model does not predict correct illusions for any of the three stimuli shown in Figure 9.

### Argyle and Snake-Illusion Variants

**FIGURE 10:**
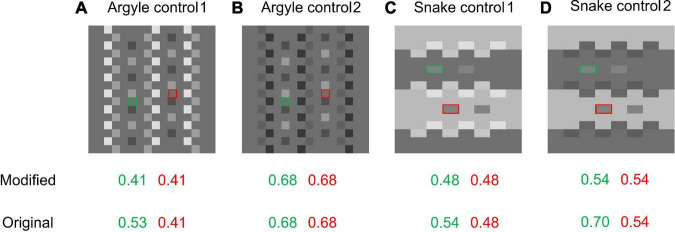
Variants of Argyle and Snake-illusion figures and the models’ predictions. Green and red frames indicate the target areas. **(A)** Argyle control 1; the darkest pixels in the original Argyle (Figure 2) are moderately brightened. **(B)** Argyle control 2; the brightest pixels are darkened. **(C)** Snake control 1; the darkest pixels in the original Snake (Figure 2) are brightened. **(D)** Snake control 2; the brightest pixels are darkened.

In this section, we show mixed results; the modified model sometimes yields better results than the original but sometimes opposite results are obtained. We thought that the modification to the prior on X-junctions would affect the models’ predictions for variants of the Argyle and Snake illusions. The appearance of the Argyle and Snake illusions is highly dependent on the X-junctions in these images, as is obvious in the “Broken Argyle” and “Snake control” images in Figure 2, where the effects are absent when the X-junctions are removed. Moreover, contrast polarities in the X-junctions are crucial for the occurrence and direction of illusory effects (e.g., Bressan, 2001). Thus, modifying the contrast polarities in the X-junctions in the Argyle and Snake-illusion figures is expected to differentiate the original and modified models’ predictions and their appearances to human observers. We created some variants of these illusions (Figure 10) and tested the models on them.

The results are shown in Figure 10. Figure 10A, or Argyle control 1, is a variant created from the Argyle image shown in Figure 2 by making the darkest regions of the X-junctions moderately bright. In this figure, the illusory effect observed in the original Argyle is almost eliminated, and our modified model predicts no illusion. However, the original model still predicts a moderate illusion and sees illuminance stripes (see the online repository for the output maps). When the X-junctions’ brightest regions in the Argyle image are made darker (Figure 10B; Argyle control 2), the illusory appearance is also eliminated, and neither model predicts an illusion.

For the variant of the Snake illusion shown in Figure 10C (Snake control 1), where the darkest regions in the X-junctions of the original Snake-illusion figure (Figure 2) are made brighter, the illusory effect becomes weaker but is still observable because of SLC. In this figure, while the original model correctly predicts a weak illusion, the modified model predicts no illusion. For Figure 10D (Snake control 2), where the brightest parts of the X-junctions are darkened, the modified model does not predict an illusion. The original model predicted an effect, but its magnitude is almost the same as, or stronger than, that for the original Snake illusion, which does not match the subjective strength of the effect.

In summary, for variants of the Argyle and Snake illusions (Figure 10), the results were mixed. The modified model shows better predictions than the original model in some cases (mainly in variants of the Argyle illusion), and the original model shows better predictions than the modified model in some cases (mainly in variants of the Snake illusion). Many more variants that can differentiate the two models’ predictions can be created, so these tests are far from exhaustive. Because further tests and comparisons with human data are needed, we cannot make a definite conclusion here on this issue.

## Discussion

In this study, we introduced a modified version of Murray’s (2020) MIR. We modified the original model in the following three aspects: (1) The belief propagation was made more efficient, (2) different potential functions are assigned for sign-variant and sign-invariant edges in X-junctions, and (3) the parameter for illuminance changes is determined flexibly based on the total luminance contrast of the input images. The first aspect is a technical improvement of the inference process, and the latter two are modifications to the model’s priors. The priors should reflect observers’ beliefs about scenes, and we believe that the two modifications largely reflect them. These “natural” priors showed significant improvements in the model’s performance without any deterioration. The main results obtained in this study are summarized in Table 2.

**TABLE 2:** Summary of the model tests performed in this study.

	Modified	Original
Checkerboard assimilation	I	W
White’s effect	C	W
Noise on SLC (Figure 4)	C	W
SCS (Figure 5A)	C	I
SCS control (Figure 5B)	C	W
Spehar’s WE variant (Figure 6A)	C	NA
Frequency’s effect on WE (Figure 7)	C	W
Target width in WE (Figure 8A)	C	W
Target height in WE (Figure 8B)	I	NA
Howe’s WE variant (Figure 8C)	C	NA
Anderson’s WE variant (Figure 9A)	W	W
Todorović’s WE variant (Figure 9B)	I	W
Clifford’s WE variant (Figure 9C)	W	W
Argyle control 1 (Figure 10A)	C	W
Argyle control 2 (Figure 10B)	C	C
Snake control 1 (Figure 10C)	W	C
Snake control 2 (Figure 10D)	W	W

*Items that were already shown in Table 1 and do not differentiate the two models’ predictions are not included here. “C” refers to correct predictions, “W” refers to wrong ones, and “I” refers to intermediate ones. WE stands for White’s effect. Given that the original model does not predict the original White’s effect, its predictions of the absence of the effect for Spehar’s variant, the target height effect, and Howe’s variant were not considered successful; thus, we marked NA instead of C.*

Specifically, the main improvements are as follows: (1) the prediction for the Checkerboard assimilation figure is closer to human perception, (2) the prediction of SLC is more stable and more immune to luminance-noise influence, (3) the predicted lightness and illuminance maps for the SCS and SCS control (Blakeslee and McCourt, 2012) are much closer to human perception, and (4) the model correctly predicts White’s effect. The first two improvements (Checkerboard assimilation and noised SLC) are largely due to the modification of the belief propagation schedule. This modification seemed to contribute to embodying the model’s assumptions in highly articulated images better. Moreover, successful predictions in the SCS variants are due to modifications to the prior about X-junctions. The original MIR is likely to interpret sign-variant edges of the X-junctions in the checkerboard as illuminance boundaries, whereas the modified MIR appropriately judges that only sign-invariant edges are cues for illuminance boundaries. The SCS control does not contain any sign-invariant edges of X-junctions, so the modified MIR correctly predicted uniform illumination. Furthermore, the correct predictions of White’s effect and its variants are mainly due to the flexible setting of the parameter for illumination changes, but the originally implemented prior—that illuminance edges tend to be straight—also plays a significant role. White-effect images tend to have high contrasts so the modified MIR expects more illumination changes, and stripes with long straight lines are likely to be judged as illuminance boundaries. These correspondences between the illusions and the added priors provide suggestions about what assumptions by humans cause these illusions.

Among the improvements we have made to MIR, the prediction of White’s effect is particularly important. It has been challenging and of high importance for lightness models because this effect highlights the fact that lightness cannot be explained solely by the contrast between adjacent areas (Wallach, 1948). White’s effect has long been known as a problem for simple contrast-based theories and has been discussed by many lightness/brightness researchers (Spehar et al., 1995; Taya et al., 1995; Anderson et al., 2001; Howe, 2005; Gilchrist, 2006; Betz et al., 2015). Although there have been various qualitative explanations of White’s effect (e.g., Howe, 2001), not many quantitative (i.e., computational) models have correctly explained it (see Blakeslee and McCourt, 1999; Lerer et al., 2021 for successful cases). Therefore, it is highly important for computational models to incorporate the prediction of White’s effect. We not only showed our model’s ability to predict this effect in a single image but also examined several variant White-effect figures. The elimination of White’s effect in double-increment/decrement conditions (Spehar et al., 1995) has rarely been predicted by computational models, but our model succeeds in predicting the presence and absence of the effect for figures whose stripe luminance levels are manipulated (Figure 6). It also predicts the effect’s enhancement caused by a higher stripe frequency (Figure 7) and in narrower targets (Figure 8A). Moreover, our model predicts a reversed effect in a variant in which targets span several stripes (Figure 8B), which matches our informal observation. Additionally, it correctly predicts the absence of the effect in Howe (2001)’s control figure (Figure 8C), which should be predicted to yield an illusion by models that depend on the processing of T-junctions (Todorović, 1997). Failures in the prediction of reverse-contrast phenomena have been a major weakness of the original MIR (Murray, 2020; Nedimović et al., 2021), but this study proved that it is possible for the MIR framework to overcome this weakness.

Although our model showed significant improvements, it still has some limitations. For example, the model is not able to account for Todorović (1997)’s illusion (Figure 9B). This may be caused by the same mechanism that is responsible for White’s effect (Gilchrist, 2006); however, our model failed to predict it. Future models should address this shortcoming by implementing additional priors, such as that on T-junctions. However, we note that none of the computational models currently available have explained all the known variants of White’s effect (Blakeslee and McCourt, 1999; Robinson et al., 2007; Zeman et al., 2015). Other complete reverse-contrast phenomena (Bressan, 2001; Agostini and Galmonte, 2002a; Economou et al., 2015) are also problems for many computational models, including MIR, so they must be addressed in future improvements.

Although we believe that our model’s additional two priors are natural (i.e., they largely reflect natural-scene characteristics), their further clarification may be possible. We simply distinguished X-junctions into two patterns, but a more detailed evaluation of X-junctions can likely be implemented. This may improve the model’s performance for illusions related to transparency or to the Argyle and Snake illusions. Regarding the prior for the expectation of illumination changes, future studies may need to consider accumulated knowledge about illumination perception by humans (e.g., Pont and Koenderink, 2007; Murray and Adams, 2019). Although our model simply expects a monotonic increase in the illuminance change’s likelihood as the total luminance contrast increases, a more complex relationship between the two variables may better reflect the illumination interpretation of humans. Incorporating “natural” priors will enhance MIR’s performance. In this study, we proposed one possible modification to MIR, but it requires further studies and improvements.

To our knowledge, this study is the first extension of Murray (2020)’s original MIR. This study highlights MIR’s high extensibility and potential; the MIR framework welcomes various patterns of modifications based on natural-scene statistics and knowledge about human vision. We improved the model’s performance by implementing two additional priors and optimizing the inference. In future studies, perceptual grouping—which is known to be an important factor for reverse contrasts (Gilchrist et al., 1999; Agostini et al., 2020)—should be implemented into the model. It may not be simple to represent perceptual grouping in CRFs, but computational methods that represent perceptual grouping, which have recently been presented, may be useful (Froyen et al., 2015; Lezama et al., 2016). These recent developments of computational methods should be highly beneficial for the MIR framework, computational models, and rigorous understanding of human vision.

## Data Availability Statement

The codes and stimuli used in this study can be found in online repositories. The names of the repository/repositories and accession number(s) can be found below: https://osf.io/ank4r/.

## Author Contributions

YK proposed and built the model through discussions with AK, collected the data, and drafted the manuscript. AK helped to construct the model and to draft and revise the manuscript. Both authors contributed to the article and approved the submitted version.

## Conflict of Interest

The authors declare that the research was conducted in the absence of any commercial or financial relationships that could be construed as a potential conflict of interest.

## Publisher’s Note

All claims expressed in this article are solely those of the authors and do not necessarily represent those of their affiliated organizations, or those of the publisher, the editors and the reviewers. Any product that may be evaluated in this article, or claim that may be made by its manufacturer, is not guaranteed or endorsed by the publisher.

## Footnotes

1 Clifford and Spehar (2003) was a conference presentation, and their original figure was not available to us. We designed the stimulus based on Robinson et al. (2007).
