# Supplementary material for: Simple Assumptions to Improve Markov Illuminance and Reflectance
Source: Front Psychol. 2022 Jul 8;13:915672. doi: 10.3389/fpsyg.2022.915672 (PMC9305333; doi:10.3389/fpsyg.2022.915672)
Supplement: Supplementary file 1 [file Presentation_1.pdf]

Supplementary Material

1 Open sources

We implemented the original MIR and our modified version in two languages: Python and Julia (https://osf.io/ank4r). They are both based on Murray (2020)'s MATLAB code. Both Python and Julia are free, so anyone can test Murray (2020)'s model and our models using them. The Python version may be familiar to more users, but the Julia version runs much faster. The repository also contains the stimuli we employed and the results of model tests. The tests were performed with the Julia version, and their results are shown in the notebook files. See the README in the repository for more details.

2 Modification of message passing

Here, we provide details about the modification we made to belief propagation. MIR adopts belief propagation using a Bethe cluster graph for inference. MIR's graph is composed of 1-nodes and 4-nodes; the former represent each node in the CRF hidden layers (i.e., the illuminance of each image pixel), and the latter represent the potential functions for each 4-clique composed of four 1-nodes. Supplementary Figure 1 (a) shows an illustration of MIR's cluster graph.

In MIR's belief propagation, messages (information about the probability of possible states of each node) are passed in vertical and horizontal trees. Message passing in trees is illustrated in Supplementary Figure 1 (b). In vertical trees, messages start at the 1-nodes in the top row, are passed to neighboring 4-nodes on the southeast (except in the leftmost vertical tree), and then are passed to 1-nodes on the southwest, and these steps are repeated. Once messages arrive at the bottom, backward passing starts, in which messages go through the same trees in reverse order, from bottom to top. Likewise, in horizontal trees, messages start at the 1-nodes in the leftmost column, are passed to 4-nodes on the northeast, and then to 1-nodes on the southwest, and these steps are repeated (except for messages in the top horizontal tree). Once messages arrive at the right end, backward passing from right to left starts. For a 16×16-sized image, messages are passed through 32 trees (i.e., 16 vertical and 16 horizontal trees), and these 32 passing patterns are randomly ordered.

In this belief propagation, most of the 4-nodes—except for those in the rightmost column or the bottom row—do not exchange messages with their neighboring 1-nodes on the southeast, as shown in Supplementary Figure 1 (b). To utilize all the links, we added one more phase of message passing after the original passing [Supplementary Figure 1 (c)]. In this additional second phase,

messages in vertical and horizontal trees go in directions that are different from those in the original phase [Supplementary Figure 1 (b)], except in the columns and rows on the CRF's edges. In most of the vertical trees, messages start from the top 1-nodes to the 4-nodes on the southwest, whereas they were sent to the 4-nodes on the southeast in the original passing phase. Similarly, in most of the horizontal trees, messages start from the leftmost 1-nodes to 4-nodes on the northeast, while they were sent to the southeast in the original phase. In the additional phase, the passing order of the 32 trees also is randomized. The additional phase starts after completing the original passing phase.

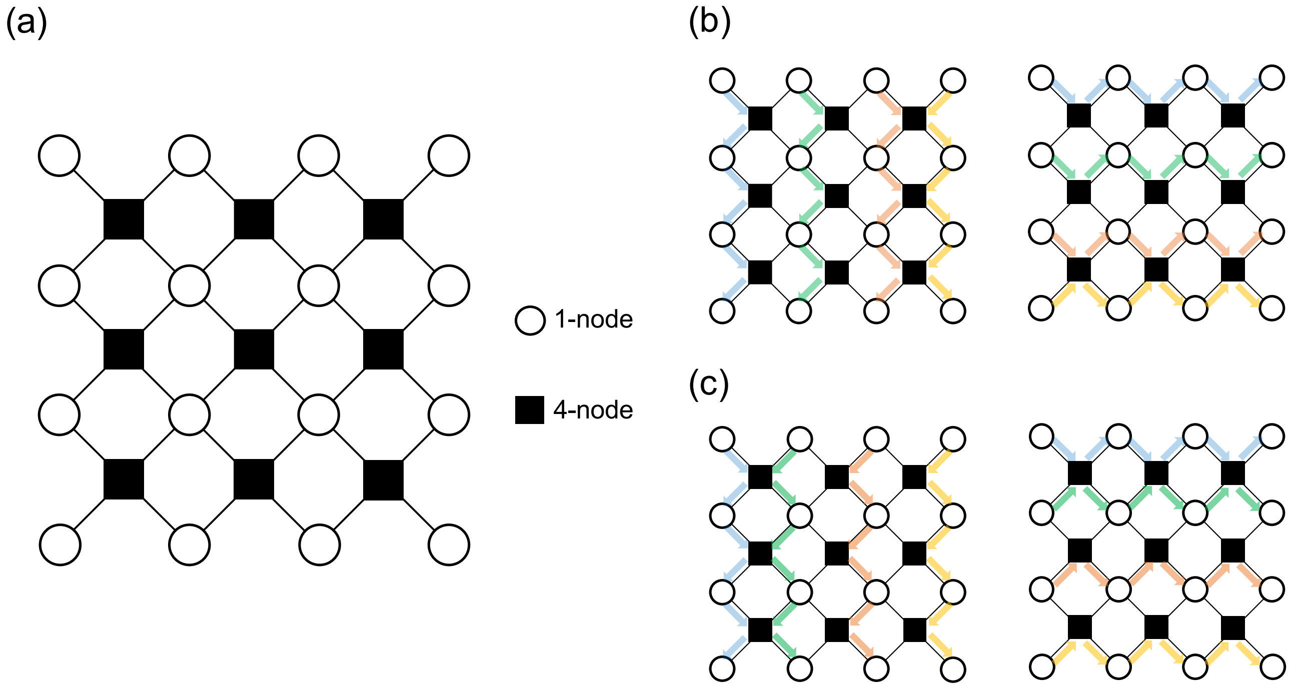

Supplementary Figure 1. (a) Illustration of MIR's cluster graph. This example shows a cluster graph of a 4 x 4 image. (b) The original message-passing process through vertical (left) and horizontal (right) trees. Arrows illustrate the message directions in forward passing. Messages go in the reverse direction in backward passing. (c) Message passing in vertical (left) and horizontal (right) trees in the additional second phase.

3 Stability of the model outputs

MIR's outputs show slight variations because its inference process is not guaranteed always to converge to one optimal solution. In accordance with Murray (2020), we reported the best results (outputs with the lowest energy) of several independent runs in the main text, but the variations of the outputs also can be useful for indicating the (in)stability of the model structure and the efficiency of the solution search. Supplementary Figure 2 (a)–(d) show violin plots of the illusion magnitudes predicted by the modified and original MIRs in the tests described in this paper. Each plot is

composed of the outputs of 30 independent runs. The best results are also plotted using circles and diamonds. Note that the best results should be treated as representative predictions and that the averages or modes of the distributions are not necessarily appropriate as representative values.

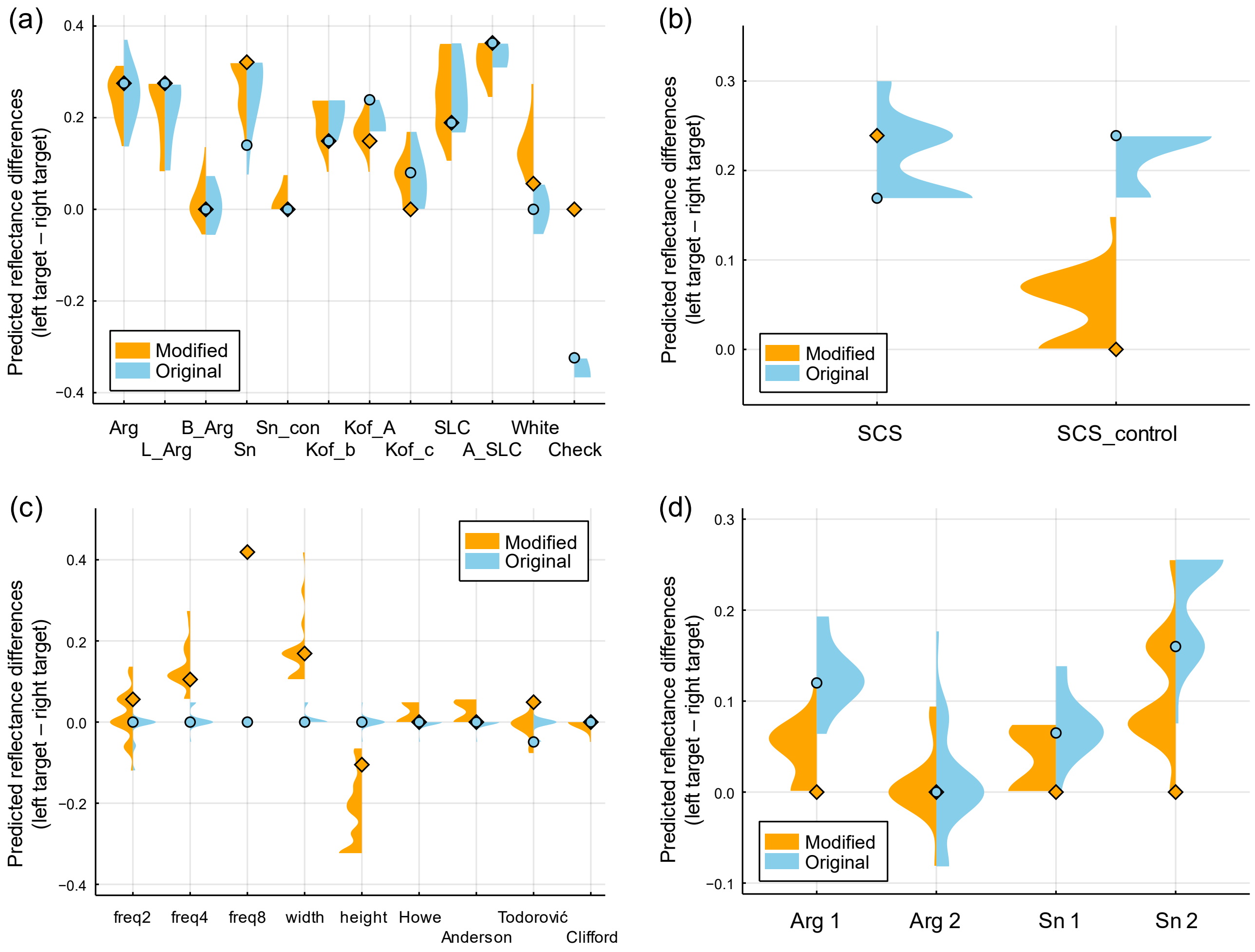

Supplementary Figure 2. Violin plots of the predicted illusion magnitudes in each model test. Each half violin is composed of 30 outputs. The orange and light-blue halves of the violins indicate the predictions of the modified and the original MIR, respectively, and the orange diamonds (modified) and light-blue circles (original) indicate the best predictions (i.e., the output with the lowest energies). The absence of violins in some conditions [e.g., Original in Sn_con in (a)] indicate that all 30 runs showed the same predictions. (a) Results of tests on the 12 original images employed by Murray (2020). The stimuli on the x-axis are in the same order as in Table 1. (b) Results of tests on the simplified checkershadow illusions. (c) Results of tests on the White-effect related figures. The stimuli on the x-axis are in the same order as shown in Figures 7–9. (d) Results of tests on the variants of the Argyle and Snake illusions.
